# Changes of T-lymphocyte subpopulation and differential expression pattern of the T-bet and GATA-3 genes in diffuse large B-cell lymphoma patients after chemotherapy

**DOI:** 10.1186/s12935-014-0085-9

**Published:** 2014-12-24

**Authors:** Qingsong Yin, Lin Chen, Qianyu Li, Ruihua Mi, Yufu Li, Xudong Wei, Yongping Song

**Affiliations:** Henan Cancer Hospital, Henan Institute of Hematology, and Cancer Hospital of Zhengzhou University, Zhengzhou, Henan China

**Keywords:** Diffuse large B cell lymphoma, Th1/Th2, T-bet, GATA-3, Immunoregulation

## Abstract

**Background and objective:**

T cell-mediated immunity plays an important role in enhancing antitumor response.This study aimed to investigate the changes in the T-lymphocyte subpopulation and to characterize the differential expression pattern of corresponding regulatory genes in peripheral blood mononuclear cells (PBMCs) from diffuse large B cell lymphoma (DLBCL) patients before and after chemotherapy.

**Methods:**

A total of 56 DLBCL patients were recruited for analysis of T-cell subset distribution in the peripheral blood using flow cytometry; serum interferon (IFN)-γ and interleukin (IL)-4 levels using enzyme-linked immunosorbent assays; and early growth response protein 1 (EGR-1), T-bet, GATA-binding protein 3 (GATA-3), and transforming growth factor (TGF)-β mRNA levels using quantitative reverse-transcription polymerase chain reaction. Twenty-six healthy subjects served as controls.

**Results:**

The percentage of CD3^+^CD4^+^T lymphocytes in peripheral blood from DLBCL patients was significantly decreased, whereas the percentages of CD3^+^CD8^+^T and CD4^+^CD25^+^T cells were significantly increased compared to those in controls (p < 0.05). Serum levels of IFN-γ and IL-4 were also significantly lower in DLBCL patients than those in controls (p < 0.05), and the levels of EGR-1, T-bet, and GATA-3 mRNA in PBMCs were lower (2.69 ± 1.48, 9.43 ± 2.14, and 20.83 ± 9.05 fold, respectively) in DLBCL patients than those in controls. Furthermore, there was a positive association between the levels of EGR-1 and T-bet mRNA (p = 0.001). However, the level of TGF-β mRNA was significantly increased in DLBCL patients, which was inversely associated with the T-bet mRNA level (p = 0.008), but positively associated with the percentage of T regulatory cells in PBMCs (p = 0.011). After three cycles of chemotherapy, the distribution of T-lymphocyte subsets in DLBCL patients were changed, and the levels of EGR-1, T-bet, and GATA-3 mRNA were significantly increased (p < 0.05) compared to those before chemotherapy.

**Conclusions:**

These results demonstrate the changes in T-lymphocyte subpopulations and the altered expression 34 pattern of the corresponding regulatory genes in PBMCs from DLBCL patients after chemotherapy, which are associated with the response of patients to treatment. The preferential expression of the T-bet gene after chemotherapy was closely correlated with the increased expression of the EGR-1 gene and decreased expression of the TGF-β gene.

## Background

Diffuse large B cell lymphoma (DLBCL) is the most common type of non-Hodgkin lymphoma in adults and is an aggressive malignancy that can arise in any part of the body [1]. At present, the standard treatment for DLBCL is chemotherapy and combination treatment with an antibody-targeted therapy. Most patients do experience good survival with a curable rate of more than 70% [2]. The precise mechanism of the development of DLBCL is not yet clear. However, it is accepted that the reason is a synergistic effect of extrinsic and intrinsic factors, and the switch in the immune state is an explanation for the development of DLBCL that has received great attention [3-5]. In the normal physiological state, B lymphocytes regulate the humoral immunity, a vital part of the adaptive immune system in the human body. Upon pathogen invasion, specific T lymphocytes will proliferate or differentiate to effector or memory T cells. Helper T cells (i.e., CD4^+^T cells) then activate specific B cells to produce antibodies for the inhibition or elimination of pathogens. Thus, CD4^+^T cells play a central role in immune protection by promoting B cell secretion of antibodies. Unfortunately, the function of B-lymphocytes is seriously deficient in DLBCL patients, which leads to abnormal humoral immune response [6-8]; similarly, the cellular immunity is extremely impaired [5,9-13].

A previous study identified two subsets of CD4^+^T lymphocytes based on cytokine production and function [14]. To date, it was found that naive CD4^+^ T helper cell (Th) precursors can differentiate into functionally distinct T cell lineages at least including Th1, Th2, Th17, and T regulatory (Treg) cells upon activation [15]. Among the critical signals that direct the induced patterns of gene expression in maturing helper T cell subsets are cytokine-induced specific transcription factors. IL-12 drives Th1 cell differentiation through the activation of the transcription factors STAT4 and T-bet [16,17], and Th1 cells mainly secrete several cytokines, such as IL-2 and IFN-γ to meditate cellular immunity. IL-4 induces Th2 cell differentiation through the actions of STAT6 and GATA-3 [18], and Th2 cells can produce the immunological factors (such as IL-4 and IL-10) to stimulate B-cell proliferation and antibody production. Whereas Th17 cells differ from Th1 and Th2 cells, whose development is prompted by a combination of IL-6 plus TGF-β and requires expression of STAT3 and the RAR-related orphan receptor gamma t (RORγt) [19], and mainly meditate inflammatory reactions, autoimmune diseases, and transplantation rejections, and are closely related to Treg cells. Treg cells negatively regulate immune function in the anti-inflammatory reaction and maintenance of immune tolerance by secreting cytokines (e.g., IL-10 and TGF-β). TGF-β is a pleiotropic cytokine, involved in many different critical processes, such as embryonal development, cellular maturation and differentiation, wound healing, and immune regulation. Paradoxically, in cancer, TGF-β has been demonstrated to be a potent inhibitor of cell proliferation and acts as a tumor suppressor at the beginning of tumorogenesis. However, once the cells become resistant to TGF-β, it mainly supports tumor growth and metastasis by promoting immune evasion and angiogenesis [20]. The number and function of these cell types are essential to maintain the balance of the immune system *in vivo*, especially, in the cross regulation and interrestraint between Th1 and Th2 cells, which can be indirectly detected by analysis of the characteristic cytokines [15]. For example, IFN-γ and IL-4 are representative and important stimulating factors that induce differentiation of Th1 and Th2 cells, respectively, and consequently indicate the dynamic equilibrium between Th1 and Th2 cells. Furthermore, T-bet and GATA-3 are important transcription factors in regulating Thl/Th2 cell differentiation. T-bet over-expression induces differentiation into the Th1 lineage, whereas loss of T-bet expression induces the cells’ default commitment to Th2 and Th17 lineages, resulting in impaired Th1 immunity. In contrast, knockdown of GATA-3 expression prevents the cells from differentiating into the Th2 lineage and over-expression in Th1 cells switches their polarity to a Th2 phenotype [21,22]. EGR-1 is a multifunctional nuclear transcriptional factor that belongs to a family of early response genes. It induced by pre-TCR signaling has been found to be important in regulation of the early stages of T-lymphocyte development, especially for the CD4^−^CD8^−^ double negative (DP) to the CD4^+^CD8^+^ double positive (DP) transition [23]; in addition, recent studies have also described EGR-1 as a tumor repressor that directly or indirectly upregulates multiple tumor suppressors, including PTEN, TP53, fibronectin, BCL-2, and TGF-β1, to inhibit cell growth, proliferation, and metastasis, as well as induce apoptosis [24,25]. Nevertheless, during B-lymphocyte tumorigenesis, the distribution of these T cells and the expression of corresponding regulatory genes could be altered [26], and detection of such alterations and gene expression patterns could be useful in monitoring DLBCL progression and treatment responses.

In our previous studies, we showed that antitumor T-cell clones exist in peripheral blood of DLBCL patients, but they do not possess effective antitumor activity [11,12], which may be due to T-cell anergy, an imbalance of T-cell subsets, or suppression of the immune system. However, little is known about the distribution of T-lymphocyte subpopulations and the expression patterns of different genes in DLBCL patients before and after chemotherapy. In the present study, we aimed to investigate the changes in T-lymphocyte subpopulations and modulation of corresponding cytokines and transcription factors (such as TGF-β, EGR-1, GATA-3, and T-bet) in PBMCs from DLBCL patients after chemotherapy.

## Results

### Redistribution of lymphocyte subsets in PBMCs from DLBCL patients

The percentage of CD3^+^T lymphocytes did not differ between healthy controls (72.90 ± 5.83%) and DLBCL patients at baseline (72.09 ± 3.16%; p > 0.05) or after three cycles of chemotherapy (71.24 ± 6.2%; p > 0.05). However, the percentage of CD3^+^CD4^+^T cells was significantly decreased (p = 0.023), and the percentages of CD3^+^CD8^+^ and CD4^+^CD25^+^T lymphocytes (p = 0.012 and p = 0.002, respectively) were all significantly increased in DLBCL patients at baseline compared to the healthy controls, resulting in a severe inversion of the CD4/CD8 ratio (Table 1). Moreover, after three cycles of chemotherapy, the percentage of CD3^+^CD8^+^T lymphocytes was significantly decreased (p = 0.015), whereas the percentages of CD3^+^CD4^+^ and CD4^+^CD25^+^T lymphocytes were increased (p = 0.095 and p = 0.009, respectively) compared to those at baseline (Table 1). The percentages of CD3^+^CD4^+^ T lymphocyte at baseline or after three cycles of chemotherapy in stage III and IV disease were lower than those in stage I and II disease, and the percentage of CD3^+^CD8^+^ and CD4^+^CD25^+^T lymphocytes at baseline or after three cycles of chemotherapy in stage III and IV disease were higher than those in stage I and II disease, yet there were no statistical significance between two groups (every p *>* 0.05).

**Table 1:** **Distribution of T-lymphocyte subsets (mean ± SE) in DLBCL patients at baseline and after three cycles of chemotherapy**

**Group**	**N**	**CD3** ^**+**^ **T cells (%)**	**CD3** ^**+**^ **CD4** ^**+**^ **T cells (%)**	**CD3** ^**+**^ **CD8** ^**+**^ **T cells (%)**	**Ratio of CD4** ^**+**^ **/CD8** ^**+**^	**CD4** ^**+**^ **/CD25** ^**+**^ **T cells (%)**
DLBCL patients at baseline	56	72.09 ± 3.16	32.31 ± 3.65	37.53 ± 6.46	0.85 ± 0.49	5.48 ± 4.96
DLBCL after three cycles of chemotherapy	56	71.24 ± 6.2	34.09 ± 4.63	32.51 ± 5.25	1.06 ± 0.87	8.32 ± 3.54
Healthy controls	26	72.90 ± 5.83	41.78 ± 4.23	28.51 ± 2.14	1.47 ± 0.27	3.68 ± 1.03

### Identification of PCR amplification fragments

**Figure 1:**
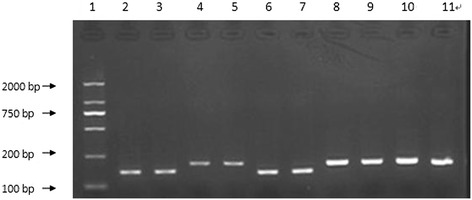
**Electrophoresis of PCR-amplified fragments.** The fragment lengths of amplification products were 159, 204, 160, 203, and 207 bp. 1, 100-bp marker; 2 and 3: T-bet; 4 and 5: GATA-3; 6 and 7: EGR-1; 8 and 9: TGF-β; 10 and 11: GAPDH.

**Figure 2:**
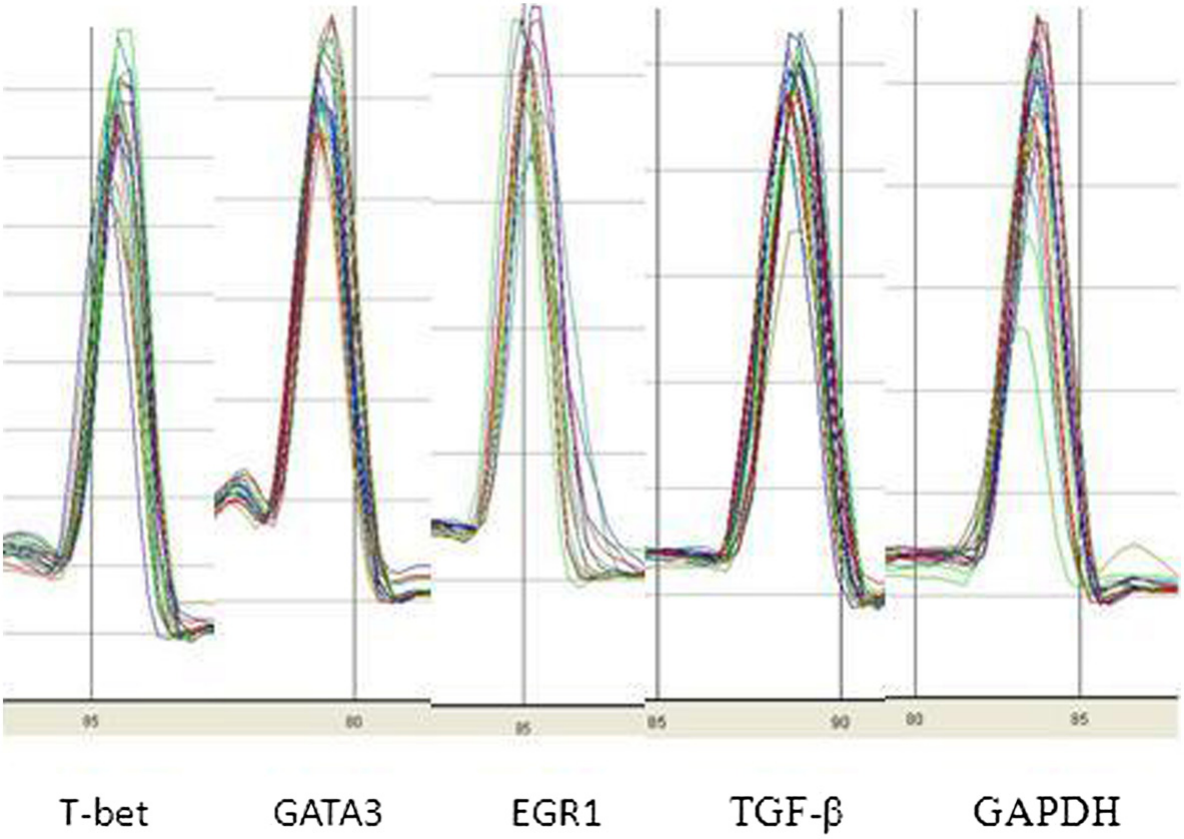
**qPCR detection of the peaks of melting curves for each transcript.** The peaks of the melting curves of PCR products for T-bet, GATA-3, EGR-1, TGF-β, and GAPDH reference gene were at 84°C, 86°C, 79°C, 85°C, and 89°C, respectively.

The amplification efficiency for EGR-1, T-bet, GATA-3, and TGF-β mRNA was consistent with that of the reference gene GAPDH. All of these transcripts were detected in all of the PBMC samples from the healthy controls and DLBCL patients. The peaks of the melting curves of PCR products for GAPDH mRNA occurred at 84°C. The peaks of the melting curves for T-bet, GATA-3, EGR-1, and TGF-β mRNA were at 86°C, 79°C, 85°C, and 89°C, respectively (Figure 1). All PCR products were confirmed using 2% agarose gel electrophoresis (Figure 2), and the results showed that the amplified fragments of T-bet, GATA-3, EGR-1, TGF-β, and GAPDH were 159, 204, 160, 203, and 207 bp, respectively, and were consistent with the expected sizes.

### Altered levels of EGR-1, T-bet, GATA-3, and TGF-β mRNA in PBMCs from DLBCL patients at baseline

**Figure 3:**
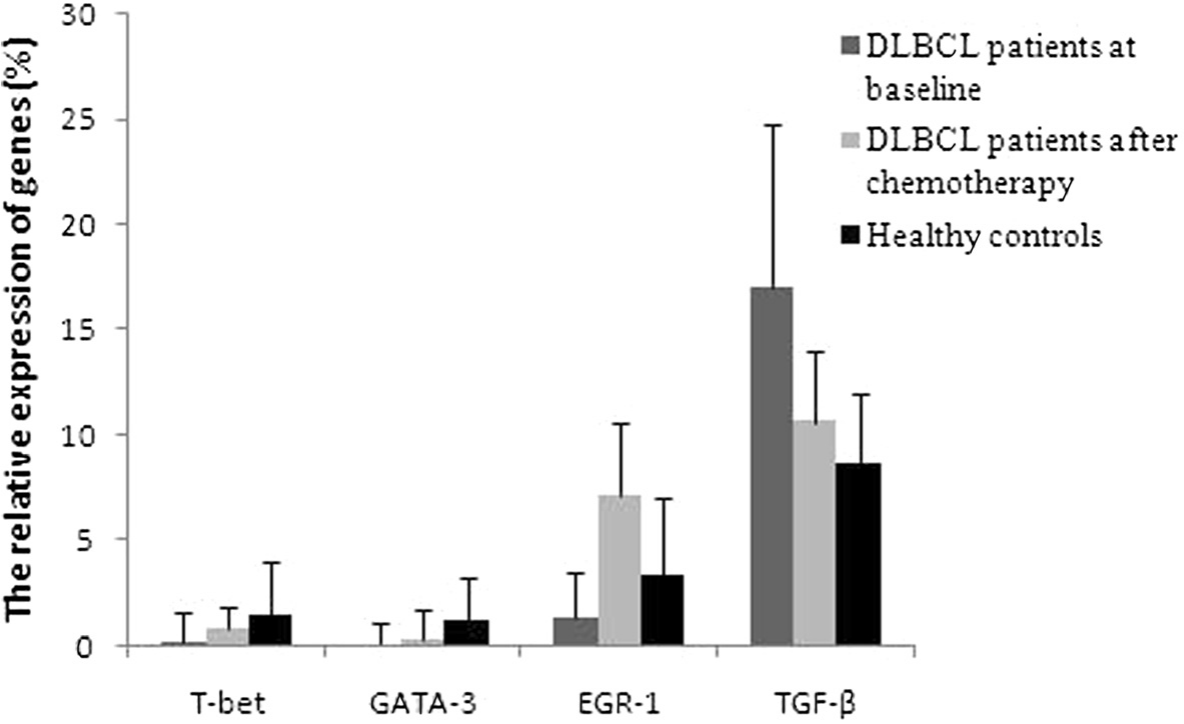
**Relative expression levels of EGR-1, T-bet, GATA-3, and TGF-β mRNA in DLBCL patients before and after three cycles of chemotherapy.** Gene expression was detected using qRT-PCR.

**Figure 4:**
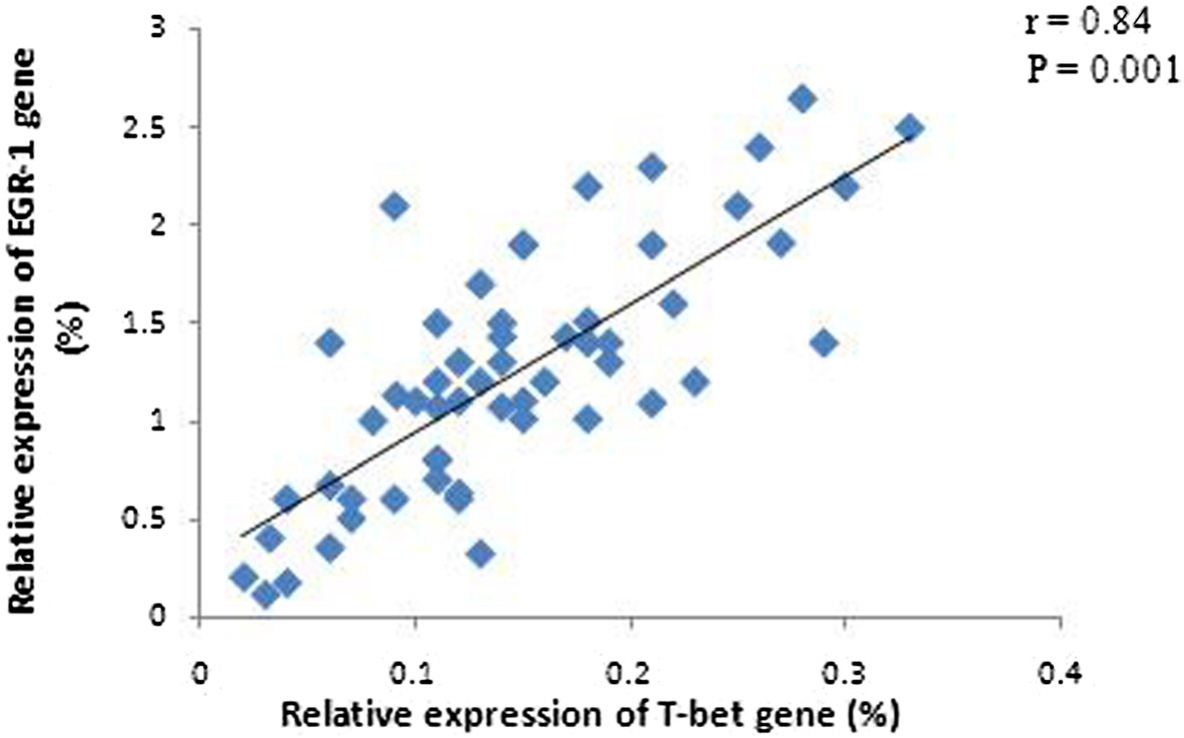
**Association of the relative expression levels of EGR-1 and T-bet genes in PBMCs from DLBCL patients at baseline.** There was an obvious positive association between the expression levels of EGR-1 and T-bet mRNA. Gene expression was detected using qRT-PCR.

**Figure 5:**
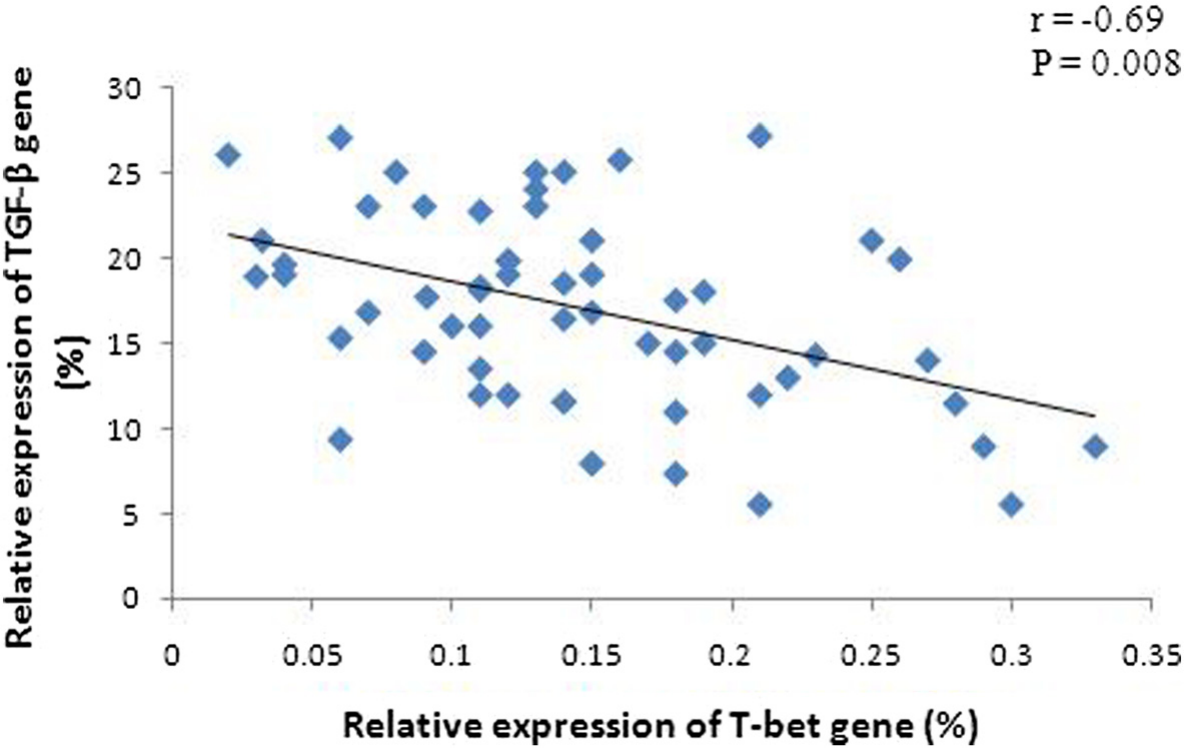
**Association of the relative expression levels of TGF-β and T-bet genes in PBMCs from DLBCL patients at baseline.** There was an obvious negative association between the expression levels of TGF-β and T-bet mRNA. Gene expression was detected using qRT-PCR.

**Figure 6:**
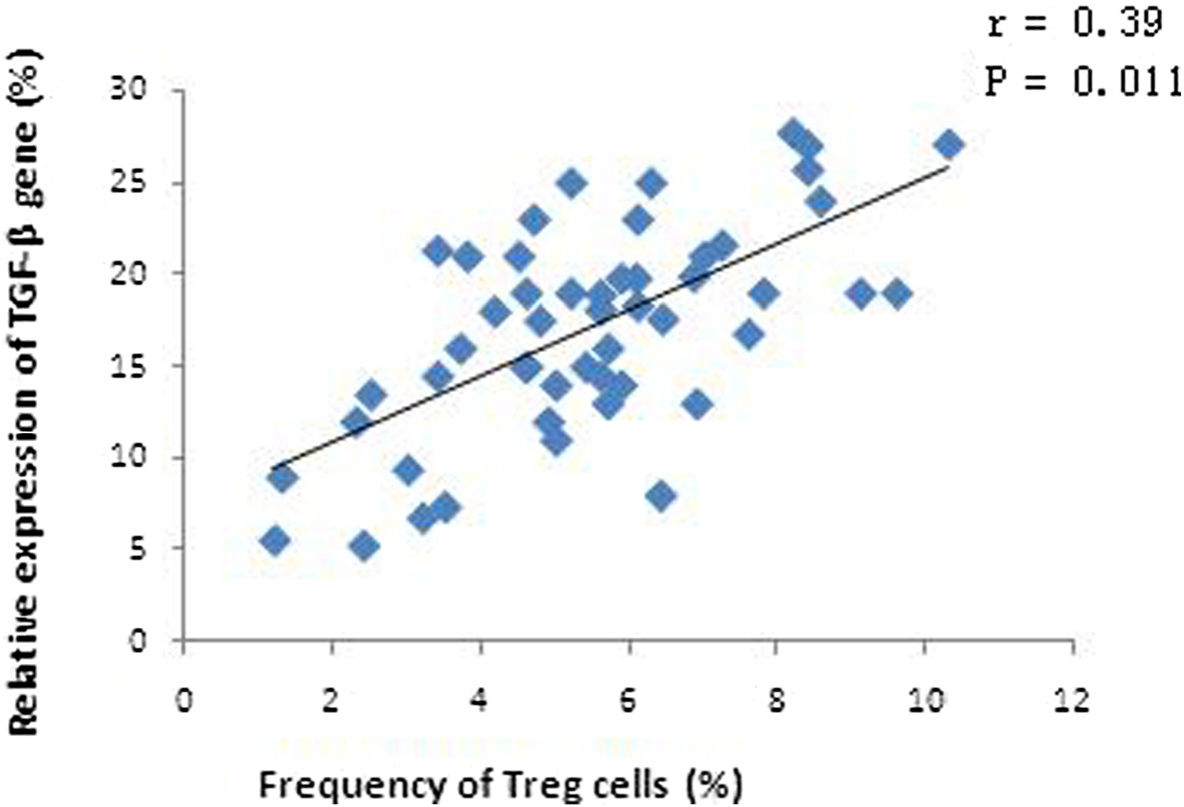
**Association of the relative expression levels of TGF-β genes and the percentage of Treg cells in peripheral blood from DLBCL patients at baseline.** There was an obvious positive association between the expression levels of TGF-β and the percentage of Treg cells. Gene expression was detected using qRT-PCR, and Treg cells were sorted by flow cytometry.

The levels of EGR-1, T-bet, and GATA-3 mRNA in PBMCs from DLBCL patients at baseline were significantly lower than those from the healthy controls (p < 0.05; Figure 3). Specifically, the EGR-1 mRNA level was decreased by 2.69-fold (±1.48), the T-bet mRNA level was decreased by 9.43-fold (±2.14), and the GATA-3 mRNA level was decreased by 20.83-fold (±9.05). Furthermore, there was a positive association between the levels of EGR-1 and T-bet mRNA in PBMCs from patients before chemotherapy (r = 0.84, p = 0.001; Figure 4). However, the level of TGF-β mRNA was higher by 2.01-fold (±0.51) in baseline PBMCs from DLBCL patients than that in the healthy controls. Furthermore, the levels of T-bet and GATA-3 mRNA in stage III and IV disease were lower than those in stage I and II disease, although there was no statistical significance (p > 0.05). The levels of EGR-1mRNA in stage III and IV disease were significantly lower than those in stage I and II disease (Table 2). In contrast, the level of TGF-β mRNA was higher in stage III and IV patients than those in stage I and II patients, yet there was no significant difference between two groups (Table 2). Moreover, an inverse association occurred between the levels of T-bet and TGF-β mRNA (r = −0.69, p = 0.008; Figure 5). There was no statistical association between the levels of GATA-3 and TGF-β mRNA. In addition, the level of TGF-β mRNA was positively associated with the percentage of Treg lymphocytes in peripheral blood (r = 0.39, p = 0.011; Figure 6).

**Table 2:** **Differential gene expression between DLBCL patients with different disease stages at baseline**

	**EGR-1**		**T-bet**		**GATA-3**		**TGF-β**	
	ΔΔ**Ct** ^**1**^	**2** ^**-**ΔΔ**Ct1**^	ΔΔ**Ct** ^**1**^	**2** ^**-**ΔΔ**Ct1**^	ΔΔ**Ct** ^**1**^	**2** ^**-**ΔΔ**Ct1**^	ΔΔ**Ct** ^**2**^	**2** ^**-**ΔΔ**Ct2**^
Stage I-II	−0.120 ± 0.67	1.09 ± 0.23	−2.496 ± 0.568	5.65 ± 1.05	−3.742 ± 0.714	13.33 ± 2.65	0.723 ± 0.793	0.605 ± 0.51
Stage III-IV	−2.952 ± 0.851	7.69 ± 2.10	−4.099 ± 1.272	17.24 ± 4.23	−5.143 ± 1.632	35.71 ± 12.41	1.337 ± 0.931	0.395 ± 0.46
P	0.008*		0.068		0.144		0.092	

Note: ΔΔCt^1^ = ΔCt_target gene in control_-ΔCt_target gene in patients_,2^-ΔΔCt1^ indicates the fold decrease in the expression of target genes in patients compared to controls. ΔΔCt^2^ = ΔCt _TGF-β in patient_-ΔCt _TGF-β in control,_ 2^-ΔΔCt2^ indicates the fold increase in TGF-β expression in patients compared to controls. *indicates that there is significant difference between two groups.

### Differential expression of serum IFN-γ and IL-4 levels in DLBCL patients at baseline

**Figure 7:**
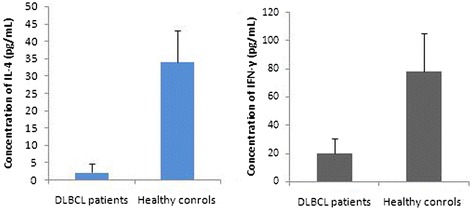
**Serum levels of IFN-γ and IL-4 proteins in DLBCL patients at baseline.** Compared to those in the healthy controls, serum levels of IFN-γ and IL-4 were significantly lower in DLBCL patients at baseline. Serum levels of IL-4 and IFN-γ proteins were detected by ELISA.

**Figure 8:**
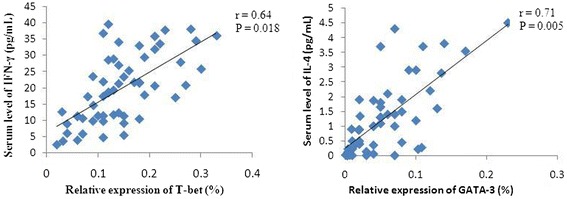
**Association of serum IFN-γ/IL-4 levels at baseline and the relative expression levels of T-bet/GATA-3 genes.** There was a positive association between serum IFN-γ level and T-bet mRNA level, yet between serum IL-4 level and GATA3 mRNA level in PBMCs. Serum IL-4 and IFN-γ levels were detected by ELISA, and gene expression was detected using qRT-PCR.

Compared to healthy controls, serum levels of IFN-γ (19.7 ± 10.5 pg/mL) and IL-4 (1.2 ± 1.4 pg/mL) in DLBCL patients were significantly lower at baseline (p = 0.000 and p = 0.001; Figure 7). Moreover, there was a positive association between serum IFN-γ level and T-bet mRNA level in PBMCs (r = 0.64, p = 0.018; Figure 8). The same was true between serum IL-4 level and GATA3 mRNA level in PBMCs (r = 0.71, p = 0.005; Figure 8). Although the serum levels of IFN-γ and IL-4 at baseline in stage III and IV patients were lower than those in stage I and II patients, there were no statistical significance between two groups (every p *>* 0.05).

### Changes of EGR-1, T-bet, GATA-3, and TGF-β mRNA levels in PBMCs from DLBCL patients after chemotherapy

**Figure 9:**
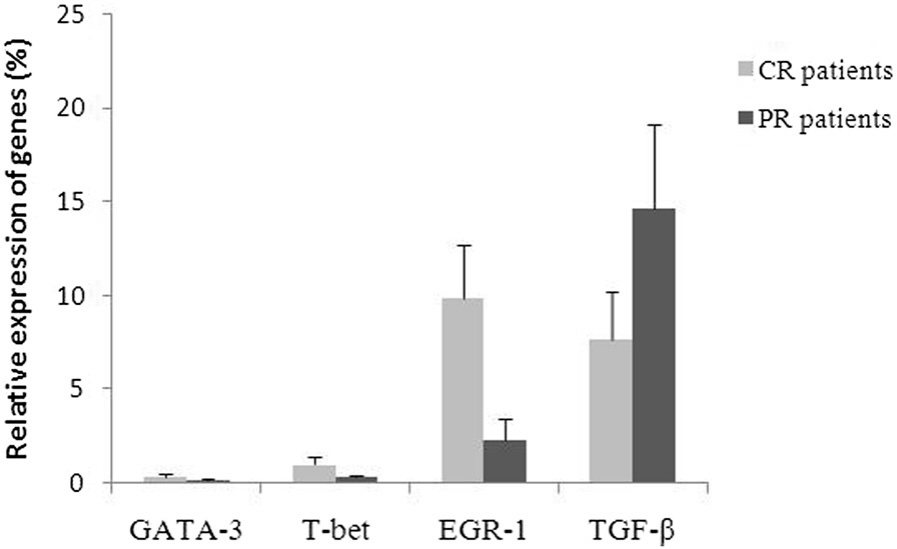
**Relative expression levels of GATA-3, T-bet, EGR-1, and TGF-β genes in CR patients and PR patients after three cycles of chemotherapy.**

After three cycles of chemotherapy, 36 DLBCL patients experienced complete remission (CR), whereas 20 DLBCL patients achieved partial remission (PR). The levels of EGR-1, T-bet, and GATA-3 mRNA were significantly higher than those before chemotherapy by 6.17-fold (±1.43), 4.84-fold (±0.95), and 3.61-fold (±1.06), respectively (p < 0.05; Table 3). EGR-1 mRNA level was the most significantly increased, reached 2.5 (±0.65) folds compared to that of healthy controls. The increase in the T-bet mRNA level was apparently higher than that in the GATA-3 mRNA level (p = 0.002). However, the levels of the two transcription factors were still lower by 1.98-fold (±1.10) and 5.71-fold (±1.62), respectively, than those in healthy controls. The levels of GATA-3, T-bet, and EGR-1 mRNA were significantly higher in CR patients than those in PR patients (p = 0.025, p = 0.005, and p = 0.002, respectively; Figure 9). However, the level of TGF-β mRNA was still higher than that in healthy controls by 1.24-fold (±0.57), although the chemotherapy reduced the TGF-β mRNA level by 1.6-fold (±1.04). In addition, the TGF-β mRNA level was lower in CR patients than in PR patients (p = 0.013; Figure 8).

**Table 3:** **Differential gene expression in DLBCL patients before and after chemotherapy**

	**EGR-1**		**T-bet**		**GATA-3**		**TGF-β**	
	ΔΔ**Ct** ^**1**^	**2** ^**-**ΔΔ**Ct**^	ΔΔ**Ct** ^**1**^	**2** ^**-**ΔΔ**Ct**^	ΔΔ**Ct** ^**1**^	**2** ^**-**ΔΔ**Ct**^	ΔΔ**Ct** ^**2**^	**2** ^**-**ΔΔ**Ct**^
Before chemotherapy	−1.427 ± 0.565	2.67 ± 1.48	−3.236 ± 1.667	9.43 ± 2.14	−4.388 ± 0.839	20.83 ± 9.05	1.001 ± 0.943	2.01 ± 0.51
After three cycles of chemotherapy	1.117 ± 0.644	0.44 ± 0.23	−0.987 ± 0.606	1.98 ± 1.10	−2.517 ± 0.796	5.71 ± 1.62	0.314 ± 0.268	1.24 ± 0.57
p	0.001*		0.002*		0.028*		0.095	

Note: ΔΔCt^1^ = ΔCt _target gene in control_-ΔCt _target gene in patients_, 2^-ΔΔCt1^ indicates the fold decrease in the expression of target genes in patients compared to controls. ΔΔCt^2^ = ΔCt _TGF-β in patient_-ΔCt _TGF-β in control,_ 2^-ΔΔCt2^ indicates the fold increase in TGF-β expression in patients compared to controls. *indicates that there is significant difference between two groups.

## Discussion

Accumulating research findings led to the conclusion/suggestion that there are multiple antitumor T-cell clones in the peripheral blood of DLBCL patients, but these T lymphocytes fail to retain effective antitumor properties [11,12]. The reason remains to be determined, but it may be due to the T-cell immune anergy and tumor immune escape resulting from imbalance of T-lymphocyte subsets. Indeed, our previous study showed that imbalance T-lymphocyte subsets occurs in PBMCs of DLBCL patients during different chemotherapy stages, and these T-lymphocyte subsets gradually return to normal by 3 months after chemotherapy [27]. CD4^+^T cells are critical in the specific and nonspecific immune response. Under normal circumstances, immature CD4^+^ T (Th0) cells differentiate into certain mature subtype for specific immune response by antigen and cytokine stimulation. Both IFN-γ and IL-4, representative cytokines secreted by Th1 and Th2 cells, are main factors that stimulate and induce Th0 cell differentiation into Th1/Th2 cells. In the current study, we found that the percentage of CD4^+^ T cells was strikingly decreased in PBMCs from DLBCL patients, whereas the percentage of Treg cells was unusually high, resulting in few cytokines secreted by Th1 and Th2 cells, the imbalance of which could always be assessed by the expression levels of these two cytokines [28].

Furthermore, both T-bet and GATA-3 are key transcription factors in regulating Th1/Th2 cell differentiation; thus, their expression directly affects the dynamic balance of the Th1/Th2 subpopulations. A previous study demonstrated that decreased T-bet expression and increased GATA-3 expression occurred in PBMCs of patients with different solid tumors [29], whereas increased T-bet expression and decreased GATA-3 expression were found in aplastic anemia patients [30]. However, to date, their expression levels are unknown in PBMCs from DLBCL patients. Our current data revealed that the levels of these two transcription factors were significantly lower in DLBCL patients than those in healthy controls, suggesting that there were few Th1 and Th2 cells in the peripheral blood of DLBCL patients. Their expression levels were even lower in DLBCL patients with stage III or IV disease than in those with stage I or II disease, which also suggests that patients with disseminated lesions have further decreased immune function and antitumor invasion capacity compared to patients with localized lesions. After three cycles of chemotherapy, the levels of T-bet and GATA-3 mRNA had increased significantly. However, it remains to be further investigated whether the increased levels of T-bet and GATA-3 mRNA contribute to the restoration of Th1 cell immune function.

Effective immune responses against different antigens are dependent on T-cell differentiation. T-bet, one of the specific transcription factors, is vital in all three important differentiation pathways of Th1 cells, i.e., TCR/EGR1, INF-γ/STAT-1, and IL-12/STAT-4 signaling pathways. A low level of INF-γ is needed for the initial activation of T-bet, which intensely stimulates the Tbx21 gene and promotes expression of T-bet through the INF-γ/STAT-1 signaling pathway [31]. Thus, the balance of T-bet and GATA-3 gene expression should be appropriately modulated during treatment, especially in elderly patients with disseminated lesions. IFN-γ intervention not only directly inhibits tumor growth but also improves *in vivo* T-bet expression after chemotherapy to promote the early reconstruction of Th1 cells and enhance antitumor immune responses. Most importantly, the TCR/EGR-1 pathway strikingly induces T-bet expression compared to the other two signaling pathways [32]. Notably, over-expression of EGR-1 through binding of EGR-1 to T-bet promoter transactivates, in concert with TCR signaling, and synergistically induces T-bet expression [33]. In the current study, we found that the level of EGR-1was reduced in DLBCL patients and was even lower in patients with disseminated lesions than that in patients with localized lesions. Interestingly, the level of EGR-1 mRNA was significantly increased by more than 2-fold in DLBCL patients after three cycles of chemotherapy. Meanwhile, T-bet expression was also apparently increased, and the increased level of T-bet mRNA was significantly higher than that of GATA-3 mRNA, suggesting that EGR-1 was positively associated with T-bet expression.

Indeed, Th1 plays an important role in enhancing the response in cell-mediated immunity. In contrast, the effects of Th2 cells mediate humoral immunity. Thus, these two types of cells are essential in maintaining the balance of the immune system. In the current study, we tried to understand the levels and balance of Th1/Th2 cells in PBMCs from DLBCL patients by analyzing corresponding transcription factors and cytokines in DLBCL patients. Our data showed that the levels of Th1 and Th2 cells were significantly lower in DLBCL patients than that in normal individuals, which will inevitably cause deficiencies in cellular and humoral immune function in DLBCL patients. In addition, tolerance induction in T cells takes place in most tumors and is thought to account for tumor evasion from immune eradication. Increased expression of cytokine TGF-β from tumor cells and T cells is associated with the occurrence, development, and prognosis of tumors by promoting tumor progression and inhibiting the immune response and surveillance system *in vivo* [34-37]. Our current study demonstrated that over-expression of TGF-β mRNA in PBMCs from patients at baseline was positively associated with a higher percentage of Treg cells, the increase of which was related to the patient’s immune tolerance and tumor immune escape. The level of TGF-β mRNA was reduced significantly after chemotherapy, but was still higher than that in healthy controls. TGF-β inhibition of the immune system is activated mainly through the ERK-2 pathway to affect expression of multiple transcription factors (e.g., T-bet and GATA-3), which seriously hampers Th1 and Th2 cell differentiation and promotes the generation of Foxp3(+) Treg cells [38,39], consistently with our current findings.

In summary, the current results demonstrate, for the first time, the changes of distribution of Th1/Th2 cells and the altered expression pattern of the corresponding regulatory genes in PBMCs from DLBCL patients after chemotherapy, which are associated with the response of patients to treatment. The preferential expression of the T-bet gene after chemotherapy was closely correlated with the increased expression of the EGR1 gene and decreased expression of the TGF-β gene. Thus, the data from the current study demonstrate that immune regulation therapy may be useful as adjuvant treatment in DLBCL patients in the future.

## Patients and methods

### Study population

A total of 56 patients (26 females and 30 males, median age 48 years, range from 24 to 72 years) were recruited from the Affiliated Cancer Hospital of Zhengzhou University between March 2009 and April 2013. These patients were newly diagnosed with DLBCL based on routinely accepted clinical and laboratory criteria with histological/cytological confirmation. According to the Ann Arbor standard staging criteria, all the patients were staged by color Doppler ultrasound, computed tomography, bone marrow biopsy, and serum lactate dehydrogenase levels. None of these 56 patients showed any bone marrow involvement. Clinically, 27 patients were at stage I to II, and 29 were at stage III to IV. Nineteen patients were older than 60 years old (i.e., 9 at stage II and 10 at stage III). All patients received CHOP (25 patients), CHOP-B (12 patients), or R-CHOP (19 patients) chemotherapy regimens. CHOP chemotherapy was administered as follows: one course of chemotherapy consisting of an intravenous infusion of 750 mg/m^2^ cyclophosphamide, 50 mg/m^2^ adriamycin, 2 mg vincristine, and an oral administration of 100 mg prednisone on days 1 to 5, which was repeated every 3 weeks. For the R-CHOP regimen, 375 mg/m^2^ rituximab was infused over 4 to 6 hours on day 1 before CHOP chemotherapy was started. For the CHOP-B regimen, intravenous bleomycin (15 mg/m^2^) was added on the basis of a standard CHOP scheme. Treatment efficacy was evaluated after three cycles of chemotherapy. This study was approved by our hospital review board, and each patient signed a consent form for participation in this study.

### Collection and analysis of blood samples

Peripheral blood samples were collected from 56 DLBCL patients and 26 healthy volunteers, and blood was withdrawn twice for the patients (at baseline and before the fourth chemotherapy cycle). The blood samples were immediately processed, and 1 mL serum from each subject was frozen and stored at −20°C until use. The PBMCs were obtained using the Ficoll-Hypaque gradient centrifugation method within 1 hour after blood sample collection. Samples of 5-10 × 10^5^ PBMCs from each patient at baseline and before the fourth chemotherapy cycle were used for flow cytometric detection of T-lymphocyte subsets, and the remaining cells were stored at −70°C for analysis of cytokines and transcription factors.

### Flow cytometric (FCM) detection of T-lymphocyte subsets

Flow cytometry was used to assess the distributions of CD3^+^, CD3^+^CD4^+^, CD3^+^CD8^+^, and CD4^+^CD25^+^CD127^−^T cells in PBMCs from healthy volunteers and patients at baseline and before the fourth cycle of chemotherapy. Monoclonal anti-CD3-PECY5, CD4-FITC, CD8-PE, and CD25-PE antibodies (BD Biosciences, San Diego, CA, USA) were used for the FCM detections according to the manufacturer’s instructions. The EP-ICS2XL flow cytometer (Beckman Coulter, Brea, CA, USA) was used for acquisition and analysis of FCM data.

### ELISA detection of serum IL-4 and IFN-γ levels

Serum levels of IL-4 and IFN-γ proteins from DLBCL patients at baseline were assessed using corresponding enzyme-linked immunosorbent assay (ELISA) kits (Takara, Dalian, China) according to the manufacturer’s protocols. Briefly, 100 μL of serum samples was added to each well of microwell plates previously coated with a polyclonal anti-IL-4 or IFN-γ antibody, and the plates were incubated for 2 h at room temperature. After a wash with phosphate-buffered saline (PBS), a polyclonal biotin-conjugated IL-4 or IFN-γ antibody was added to each well, and the plates were further incubated for 1 h at room temperature. After washing, streptavidin-horseradish peroxidase (HRP) was added to each well and incubated for 1 h, and then the unbound streptavidin-HRP was washed away. Next, the color reagent for HRP was added to the wells, and the absorbance was measured at 450 nm using an Elx808IU Ultra Microplate Reader (Bio-Tek, Winooski, VT, USA). A standard curve was constructed using the IL-4 or IFN-γ protein standards provided in the kits. Each sample was analyzed in triplicate.

### RNA isolation and qRT-PCR

Total cellular RNA was isolated from 1-5 × 10^6^ PBMCs using a Trizol reagent (Invitrogen, Carlsbad, CA, USA) and then 2 μg RNA was reversely transcribed into the first single-strand cDNA using random hexamer primers and the Superscript II reverse transcriptase Kit (Invitrogen, USA) according to the manufacturer’s instructions. The RNA quality was analyzed in 0.8% agarose gel stained with ethidium bromide. The cDNA quality was confirmed by RT-PCR for glyceraldehyde-3-phosphate (GAPDH) reference gene amplification.

For the detection of EGR-1, T-bet, GATA-3, TGF-β, and GAPDH reference gene mRNA levels by SYBR Green I real-time PCR (Ex Taq II-DRR820A, Takara), the primers (Table 4) were synthesized by Shanghai SANGON Biological Engineering Technology Services Co., Ltd. (Shanghai, China). PCR amplification was performed using an ABI 7300 (Applied Biosystems, Foster City, CA, USA) in a 25 μL total volume containing 50 ng/ μL cDNA, 10 μmol/ L primer pairs, 10 μL of 2 × Real Master Mix (Ex Taq II-DRR820A, Takara), and 0.4 μL ROX (Ex Taq II-DRR820A, Takara) with an initial denaturation at 95°C for 30 s and then 40 cycles of 95°C for 15 s and 61°C for 31 s. Additionally, a specific amplification of the PCR products was analyzed by melting curve analysis and agarose gel electrophoresis. On the basis of the consistent amplification efficiency for each of target genes and GAPDH reference gene, the relative amount of the target genes was normalized to that of GAPDH mRNA in two independent assays using the 2^-△Ct^ × 100% and 2^-△△Ct^ methods [40-42].

**Table 4:** **Primer sequences for qPCR**

**Gene**	**Sequences**
GAPDH	5′-ACGGATTTGGTCGTATTG-3′
	5′-GGAAGATGGTGATGGGATT-3′
T-bet	5′-GGGCGTCCAACAATGTGA-3′
	5′-CGGCAATGAACTGGGTTT-3′
Gata-3	5′-ATGAAGGATGCCAAGAAGT-3′
	5′-TGAACAAATGATTCGCCTA-3′
EGR-1	5′-CGATGAACGCAAGAGGCA-3
	5′-CGGGGATGGATAAGAGGTAGT-3′
TGF-β	5′-GAAACCCACAACGAAATCT-3′
	5′-AGGTATCGCCAGGAAT-3′

### Statistical analysis

All statistical analyses were performed using the Statistical Package for Social Sciences software (SPSS 18.0, Chicago, IL, USA), and the data are presented as mean ± standard error (SE). Differences in mRNA levels between the two groups were analyzed using Student’s *t* test. The Spearman’s rank correlation analysis was performed to detect differences in the mRNA expression of EGR-1, T-bet, GATA-3, and TGF-β in different samples. P < 0.05 was considered statistically significant.
